# Supplementary material for: Are carbon emissions trading and green financial instruments synergistic? -Comprehensive quantitative research based on content analysis
Source: PLoS One. 2024 Mar 7;19(3):e0298601. doi: 10.1371/journal.pone.0298601 (PMC10919601; doi:10.1371/journal.pone.0298601)
Supplement: S1 Data — (ZIP) [file pone.0298601.s001.zip › Data/地级市Z_F工作报告.zip!/░▓╗╒/╨√│╟╩╨/└┤╫╘╣1⁄2╓┌║┼í╛┬φ┐╦╩2╛▌═°í┐_2019.docx]

——2019年1月9日在宣城市第四届

人民代表大会第三次会议上

宣城市人民政府市长	张冬云

各位代表：

现在，我代表市人民政府，向大会报告政府工作，请予审议，并请市政协各位委员提出意见。

一、2018年工作回顾

2018年是宣城发展进程中极不平凡的一年。我们在省委、省政府和市委的坚强领导下，以习近平新时代中国特色社会主义思想和党的十九大精神为指导，着力“对标沪苏浙、争当排头兵”，迎难而上、开拓进取、扎实工作，圆满完成市四届人大二次会议确定的目标任务，三大攻坚战初战告捷，高质量发展起步有力，皖苏浙省际交汇区域中心城市建设呈现崭新局面。

这一年，我们聚焦发展第一要务，践行新发展理念，制定实施促进经济高质量发展“1+N”政策体系和支持民营经济发展“30条”，经济运行稳中有进、进中向好。地区生产总值跨越两个百亿台阶，达1317亿元，增长8.32%，经济总量排位在全省十六个市和长三角城市经济协调会中均实现前移。财政收入240.1亿元，增长9%。规模以上工业增加值、固定资产投资分别增长11%和14.9%，均创2015年以来最高增速。社会消费品零售总额、进出口总额分别增长9.5%和21.1%。主要经济指标增幅好于预期、高于全国全省，实现了在全省“保八争七”基础上的争先进位。跻身中国城市创新竞争力和2018中国地级市全面小康指数全国前100名，创新持续力居长三角城市第3位，获评新时代中国全面建成小康社会优秀城市。入选全国12个2018美丽山水城市，系安徽省唯一。

这一年，我们以实际行动庆祝改革开放40周年，长三角一体化发展上升为国家战略，宣城发展迎来了前所未有的历史性机遇。年度108项重点改革任务顺利推进，民营经济占比居全省首位，沪宣互发全国首张异地办理营业执照，医改典型做法两次获得国务院办公厅通报表扬。正式加入长三角城市经济协调会和G60科创走廊3.0版，《苏皖合作示范区发展规划》获国家发改委批复，深度融入长三角站上了新起点。8家开发区进入《中国开发区审核公告目录(2018年版)》,居全省第4位，为打造经济增长新引擎构筑了一批高水平的合作平台。

这一年，我们坚持以人民为中心，持续增强群众获得感、幸福感、安全感。城镇、农村居民人均可支配收入达36554元和16013元，分别增长9%和9.8%，居民收入跑赢经济增长，总量均居全省第4位。顺应人民群众对美好生活的向往，办成了一批宣城老百姓期盼已久的大事。杭黄高铁和广宁高速开通运营，宣绩高铁关键工程正式开工，芜黄高速宣城段加快建设，“县县通高铁、域域通高速”的梦想即将成真，中心城区“一环”闭合通车、“二环”春节前贯通，15年一贯制的北师大宣城学校建设顺利，省立医院皖南妇幼分院主体封顶。社会大局保持和谐稳定，人民群众安全感和对公安工作满意度均居全省第一。

（一）制造业发展迈上新台阶。转型升级步伐加快。高新技术产业增加值占比首次突破50%，达54.2%；增长20.1%，居全省第4位。战略性新兴产业产值增长21%，居全省第3位。工业投资、工业用电量增幅均居全省第1位。新增国家两化融合贯标达标企业17户、绿色工厂4户和省级“专精特新”企业34户。中鼎集团被认定为中国制造业单项冠军示范企业。国家级宣纸及文房用品质检中心全面建成。泾县高效节能电机列入省重大新兴产业工程名单，皖南电机获省政府质量奖提名奖，旌德云乐灵芝被评为国家林业标准化示范企业。新认定中国驰名商标3件。成功举办中国制造强国（宣城）高峰论坛和2018中国·宣城新能源新材料产业发展高峰论坛。发展动能加速转换。省级创新型城市建设顺利推进。宁国入选首批国家级创新型县（市）。宛陵科创城一期主体竣工，近30家单位确定入驻。智谷软件产业园入驻企业84户。新建省级以上创新平台16家，获批国家级企业技术中心2家。实施“宛陵聚才行动”，招引高层次人才团队20个，入选省级团队7个，居全省第1位。研发经费投入强度增幅居全省第6位。每万就业人口中研发人员数量增长19%，居全省第1位。万人发明专利拥有量达9.5件。新认定高新技术企业96户，居全省第6位。供给体系质量提升。加快破除无效供给，处置“僵尸企业”66户；消化批而未供土地11334亩，处置闲置用地6742亩，盘活低效用地8756亩。常态化开展“四送一服”双千工程，减免企业税费8.4亿元，规模工业企业利润增长42.5%，居全省第4位。持续开展“春风行动”，举办各类招聘会623场，新招工11.7万人。金融服务实体经济力度加大，发放新型政银担贷款55亿元、税融通贷款13亿元，新增贷款155亿元。村镇银行实现县域全覆盖。华菱精工A股上市，新增省区域性股权市场挂牌企业42户，直接融资119.4亿元，创历史新高。有效防范化解金融风险，金融机构不良贷款余额、不良贷款率实现“双下降”。政府债务管理和风险管控得到加强，除省代发地方政府债券外，没有违规新增政府债务，存量债务积极化解。

（二）园区发展焕发新活力。全市开发区优化整合方案获省政府批复，广德、郎溪经开区分别加挂“皖苏浙产业合作园区”“皖苏产业合作园区”。开发区转型升级考核居全省第5位，创历史最好成绩。4家开发区进入全省前30强，居全省第1位。宣城经开区新签好彩头食品、蚂蚁乐居智慧家居等亿元以上重大项目53个，总投资110亿元；新开工工业项目46个，鸿路钢构一期等32个项目竣工投产，金能移动能源、华威铜箔等项目加快推进。宣城现代服务业产业园区发展加快，农批市场二期、冷链物流大市场一期和皖南最大的建材（钢材）物流中心基本建成，农批市场交易额达120亿元。新塘羽绒产业园落户企业40户，中国新“羽都”初步形成。宣城高新区获批省“多评合一”试点，入围G60科创走廊产业联盟重点园区，争创国家级高新区进展顺利。县域经济协调发展。宁国、宣州进入全国综合实力百强县（区）。广德跻身全国投资潜力百强县。郎溪荣获安徽省质量强县。宁国荣获3项国务院督查激励。泾县获评国家传统知识产权保护试点县。绩溪、旌德绿色产业发展壮大。

（三）现代服务业迈出新步伐。新增限上商贸流通企业43户。9户企业入选安徽省“老字号”。宣城万达广场正式开业。皖东南名品展销会首次走进长三角。广德获评中国电商示范百佳县，泾县、广德、绩溪获评全省电商进农村全覆盖工作先进县。文房四宝产业发展规划正式发布，7户企业入选安徽民营文化企业100强。国家全域旅游示范区创建扎实推进，接待游客数、旅游总收入增长16.5%和19.2%。敬亭山风景名胜区旅游设施得到提升，13个重点项目加快实施。金陵大饭店按五星级标准建设，3家旅行社获批出境社资格。建成县级旅游集散中心4个，旅游厕所、标识牌、景区“最后一公里”建设加快。发行“宣城旅游年卡”。皖南川藏线完成开发建设投资15.5亿元，入选2018中国体育旅游十佳精品旅游线路。

（四）基础设施建设取得新进展。举行四次重点项目集中开工竣工投产活动，累计开工竣工投产项目820个，总投资1400亿元以上。列入省亿元以上重点项目投资完成率、开工率、竣工率均居全省前列。市重点项目完成投资680亿元，争取中央预算内投资和省补助资金8.6亿元。昌吉—古泉±1100千伏特高压直流输电工程全线通电，绩溪抽水蓄能电站开始蓄水。省地矿局311地质队正式挂牌入驻宣城，宣州茶亭数千亿级的铜多金属矿取得重要突破。完成铁路、公路等交通建设投资101亿元。芜广铁路完成电气化改造，商合杭铁路、皖赣铁路扩能改造宣城段和芜宣机场、水阳江航道、巷口桥铁路物流基地加快建设。宁宣城际铁路规划获批。省际“断头路”全面动工。3个一级公路项目建成。高速公路和一级公路通车里程均居全省第3位。宣城火车站东站房、客运总站和宣州综合码头一期建成或使用。完成水利投资15.7亿元。列入国家重大水利工程的港口湾水库灌区、牛岭水库开工建设。青弋江治理和粮长门水库基本完工。城东联圩、双桥联圩、敬亭圩堤防加固建设加快。建设农村道路畅通工程772.5公里，泾县被命名为“四好农村路”全国示范县。新建续建输变电工程12项，竣工投产4项。建成覆盖全市的窄带物联网。

（五）城市品质得到新提升。扎实开展全国城市设计试点，推进城市设计全覆盖，入选全国城市设计经验交流城市。加强规划引领，新编、续编规划79项。市区实施畅通、安居、活水、安保、美丽“五大工程”，统筹推进“十大板块”建设，续建和新建项目167个，完成投资196亿元。21条市政道路交付使用，14条道路开工建设。“533”社会事业补短板工程全面推进，皖南医药卫生学校开工建设，市人民医院一期改扩建工程投入使用，市委党校新校区、市五中、皖南中医院等项目完成选址。全民健身馆等11个项目基本完工。实施城区5条内河水质提升工程。新增绿地面积70公顷。国家节水型城市创建通过现场考核。强化征迁保障，开展“清零”行动23次。全市完成征迁拆违174.4万平方米，其中市区100.4万平方米。依法化解北门大唐、中国茶府、利华世纪新城等历史遗留问题。房地产市场保持平稳健康发展。市区分配安置房5955套，年节约超期过渡费近7000万元。高水平通过全国文明城市年度测评，广德、宁国获2018—2020年全国文明城市提名。启动市区生活垃圾分类试点。市生活垃圾焚烧发电项目一期投产。推进城市执法体制改革，城市治理新格局初步形成。

（六）乡村振兴实现新开局。现代农业稳步发展。粮食总产134.6万吨。农产品加工产值890亿元，增长9.9%。茶叶、家禽等特色产业发展壮大。获批省级农产品质量安全县2个，新增“三品一标”产品64个。宣城现代功能农业科创园等园区加快建设，获批全国休闲农业与乡村旅游五星级园区3个。新增省级示范合作社12个、家庭农场27个、产业化示范联合体9个。农业“两区”划定如期完成。科学防控非洲猪瘟疫情，扎实开展“大棚房”问题排查整治。体制机制不断创新。被确定为全国农村集体产权制度改革整市试点单位。411个村实施农村“三变”改革。新增土地流转10.4万亩，流转率达46%。农村产权交易市场建成运营。深化林地经营权流转证制度改革。旌德颁发全国首本农村宅基地“三权分置”不动产权证书。宁国获得全国农村创业创新典型县范例。乡村面貌持续改善。美丽乡村建设和农村人居环境整治扎实推进。49个省级中心村建设进度超省定目标。整治11个非正规垃圾堆放点，建成42个乡镇政府驻地污水处理设施，改造自然村非卫生厕所4万户，行政村生活垃圾收运处理实现全覆盖。新增中国美丽休闲乡村3个、传统村落36个。精准脱贫年度任务顺利完成。推广“四带一自”“三有一网”扶贫模式，累计实施到村产业项目734个、到户10.5万个，入选全省产业扶贫十佳园区2个、十佳合作社1家。在全省率先实施贫困人口疾病保障“5090”工程。旌德在全国率先试点“智慧医疗”进村入户助力健康扶贫。困难群众帮扶工作在全省率先实现全覆盖。

（七）改革开放开启新征程。重点领域改革不断深化。“证照分离”改革全面推开，新登记市场主体3.6万户。“一网一门一次”改革取得突破，在全省率先形成覆盖市县乡村四级的“互联网+政务服务”体系、统一的“12345”政府服务热线平台，全面开展“减证便民”，政务服务事项申请材料精简58.5%，不动产登记办结时限提速90%。市属国有平台公司市场化转型步伐加快。区域一体化发展深入实施。G60科创走廊九地市共同发布“扩大开放30条政策”，建立一体化发展的开放型经济体系。秉持共建共享同城化，加快与南京都市圈城市间互联互通互融。积极谋划推进长三角产业合作示范区核心区建设。招商引资质量提升。开展招商引资“百日会战”。新建亿元以上项目363个，亿元以上项目到位省外资金885.2亿元。新设外商投资企业19户，实际利用外资11.2亿美元，增长21.7%。在首届世界制造业大会上，27个项目入选省“五个一百”集中签约项目，投资总额超过220亿元。对外贸易优化升级。皖东南保税物流中心封关运行。国际贸易“单一窗口”办理实现全覆盖。新增进出口实绩企业98户、境外投资企业7家。参加首届中国国际进口博览会，33户企业加入G60采购商联盟。

（八）生态文明建设取得新进步。以强烈的政治担当，依法依规、从严从实推进扬子鳄自然保护区生态环境问题和中央环保督察及“回头看”反馈问题整改。蓝天、碧水、净土保卫战顺利推进，PM2.5年均浓度和优良天数比例分别居全省第3位和第2位。国控断面水质达标比例全省领先，最严格水资源管理考核获省优秀等次。秸秆综合利用率、畜禽养殖废弃物资源化利用率分别达90.6%和82.4%，秸秆禁烧实现卫星监测“零火点”。河（湖）长制、林长制全面实施。林业增绿增效任务超额完成。旌德建成全国首个林长制智慧信息管理平台，绩溪在全省率先推行林区警长制和“技防河长”。全面启动水清岸绿产业优美丽长江（宣城）经济带建设，安徽（宣城）绿色发展创新实验区加快建设。

（九）社会事业补短板呈现新亮点。民生保障持续加力。33项省定民生工程、10项市政府民生实事顺利完成。城镇新增就业5.7万人，城镇调查失业率4.8%。超额完成社会保险参保任务。完成机关事业单位和离退休人员工资调整，提高最低工资标准和城乡低保、居民基本医保标准。推进社会养老服务体系建设。全市开工棚户区改造9983套，基本建成15289套。改造老旧小区51个、农村危房4030户。新解决1.85万农村居民饮水安全问题。社会事业加快发展。全市新改建公办幼儿园12所。实施高中阶段教育普及攻坚计划。民办教育、中等职业教育加快发展。启动健康城市建设，列入国家社会办中医试点地区，再获全国无偿献血先进市称号，县域医共体试点县实现全覆盖。计划生育服务管理水平持续提升。创成省级公共文化服务示范区3个，在全省率先实现农家书屋数字化全覆盖，宣城、宁国、绩溪3家图书馆创成国家一级馆。精心组织第四次全国经济普查、第三次全国国土调查工作。妇女儿童、青年、老龄、残疾人、红十字等事业进一步发展，外事侨务、对台、机关事务管理、档案、地方志、科普等工作取得新成绩。社会治理水平提升。扫黑除恶工作成效居全省第2位。综治工作连续6年、信访工作连续12年在全省获得先进位次。扎实推进“七五”普法工作，完善公共法律服务体系。健全安全生产责任体系，深入开展安全生产攻坚行动，有效处置突发事件，安全生产形势持续平稳。创成省级食品药品安全城市。民族宗教工作平稳有序。获评全国防震减灾工作考核先进单位。成立退役军人服务管理机构，蝉联全省双拥模范城，国防动员、民兵预备役和人民防空工作取得新成效。

（十）政府自身建设得到新加强。深入学习贯彻习近平新时代中国特色社会主义思想，树牢“四个意识” ,坚定“四个自信”,坚决做到“两个维护”，扎实开展“讲严立”专题警示教育，切实抓好省委巡视反馈意见整改落实。加强法治政府建设，出台两部政府规章，严格执行重大行政决策程序规定，完成新一届市政府法律顾问聘任。自觉接受市人大及其常委会的法律监督和市政协的民主监督，依法执行市人大决议决定，办理市人大代表建议95件、市政协委员提案288件。深化政务公开，整市推进基层政务公开标准化规范化试点。推进政府系统全面从严治党向纵深发展，认真落实中央八项规定及实施细则精神和省、市委有关规定，“三公”经费等一般性支出进一步压缩。启动新一轮审计监督全覆盖，开展审计（调查）项目1445个，增收节支5.3亿元。坚持真抓实干，强化经济运行、项目建设调度，狠抓政务督查督办、考核考评、问责问效。连续两年获省政府目标管理绩效考核先进单位。

看似寻常最奇崛，成如容易却艰辛。过去的一年，面对宏观环境复杂严峻、经济下行压力加大、深层次矛盾逐步显现的多重困难，面对扬子鳄自然保护区生态环境问题整改、非洲猪瘟疫情防控的艰巨任务，我们在市委的坚强领导下，在市人大、市政协的监督支持下，攻坚克难、砥砺奋进，取得的成绩实属不易，这是全市上下撸起袖子干出来的。在此，我代表市人民政府，向在各个岗位上辛勤工作的全市人民，向给予政府工作大力支持的人大代表、政协委员，向各民主党派、工商联、各人民团体和社会各界人士，向驻宣解放军指战员、武警官兵和政法公安干警，向关心、支持宣城发展的中央和省驻宣单位，向在我市投资创业的广大企业家，表示衷心的感谢和崇高的敬意！

同时，我们清醒认识到，全市经济社会发展中还存在不少困难和问题。供给侧结构性改革任务艰巨，实体经济面临的困难较多，发展质量和效益仍需提高；脱贫攻坚、污染防治、化解政府债务仍需持续发力；创新要素集聚能力不够好，新旧动能转换仍需加快；市本级辐射带动力不强，一些历史遗留问题仍需加快化解，中心城市建设任重道远；要素支撑趋紧，经济发展与能源消费总量约束的矛盾突出；经济外向水平不高，开放平台、进口短板亟待加强；社会事业和民生领域还有不少短板，与人民群众美好生活需要相比仍有较大差距；少数公职人员法治意识和服务意识淡薄，业务能力和落实水平有待提升，干事创业激情有待增强，创优“四最”营商环境还需加力。对此，我们将继续努力改进，绝不辜负全市人民的期待。

二、2019年工作安排

2019年是中华人民共和国成立70周年，是全面建成小康社会关键之年。2019年有机遇也有挑战，我们要始终把高质量发展作为解决宣城一切问题的关键，坚持以经济建设为中心不动摇，坚持新发展理念，保持战略定力，坚定发展信心，牢牢抓住用好重要战略机遇期，一起拼搏、一起奋斗，在百年未有之大变局中展示宣城新作为。

做好今年政府工作，要以习近平新时代中国特色社会主义思想和党的十九大精神为指导，深入贯彻落实中央、省委经济工作会议精神和市委四届六次、七次全会部署，坚持稳中求进工作总基调，坚持“巩固、增强、提升、畅通”八字方针，落实“六稳”工作要求，努力做到好中求快，以“对标沪苏浙、争当排头兵”为总抓手，实施“十项对标提升工程”，统筹推进稳增长、促改革、调结构、惠民生、防风险工作，保持经济持续健康发展和社会大局稳定，加快建设皖苏浙省际交汇区域中心城市，为全面建成小康社会收官打下决定性基础，以优异成绩庆祝中华人民共和国成立70周年。

统筹考虑全市经济社会发展的主要预期目标是：地区生产总值增长8%以上，财政收入与经济增长同步，规模以上工业增加值增长9%左右，固定资产投资增长10%左右，社会消费品零售总额增长9.5%左右，进出口总额增幅高于全省平均水平，城镇居民人均可支配收入与经济增长同步，农村居民人均可支配收入增长9%左右，城镇新增就业4万人以上，城镇调查失业率控制在5.5%以内，完成生态环保、节能减排年度任务。

着力做好以下八个方面工作：

（一）坚持工业强市不动摇，推动制造业高质量发展。牢固树立“抓发展必须抓产业，抓产业首在抓工业”理念，强化工业主体地位，聚焦绿色智造，以先进制造业为主攻方向，加快推进产业向中高端迈进。

强化企业培育。实施龙头企业培育工程，推动“个转企”“小升规”，新增规上企业100户以上。实施“专精特新”培育计划，培育认定省级以上“专精特新”中小企业25户，新增一批行业“单打冠军”和“配套专家”。培育省级以上两化融合贯标试点企业8户。继续实施“万家企业上云”计划，力争“上云”企业3000户以上。实施增品种、提品质、创品牌“三品”战略，培育省级新产品20个、安徽工业精品6个以上。

强化工业投资。加快鸿路钢构绿色智能制造基地、国肽大健康产业中心、陆达锂电材料、紫燕食品等重点工业项目建设，确保绩溪抽水蓄能电站首台机组并网发电，完成宁国抽水蓄能电站可研编制，力争工业投资增长20%以上。实施“千企升级”计划，完成技术改造项目220个以上，确保工业技改投资增长20%以上。实施新兴产业“双百项目”计划，新开工、投产项目240个以上。推进智能制造“915”工程，推广应用工业机器人900台，实施10个智能装备项目，打造5个智能工厂、数字化车间。

强化产业集聚。围绕培育“4+1”千亿产业，制定主导产业推进意见和个性化扶持政策，加快培育汽车及装备制造、新能源新材料、食品医药、电子信息等主导产业，着力打造具有核心竞争力的产业集群。推进“三重一创”建设，高水平建设省、市战略性新兴产业基地和泾县高效节能电机新兴产业工程，确保战略性新兴产业产值增长12%左右。加快培育“四新”经济，大力发展数字经济，加快智谷软件产业园等“双创”基地建设，着力培育“互联网+制造”示范。

强化园区发展。深化开发园区管理体制机制改革，建立评价考核体系，推进建设和运营模式创新。力争更多开发区进入全省30强。支持广德、郎溪经开区争创国家级开发区。完善园区产业配套体系和综合服务功能，促进园区产业集聚，支持各开发园区确定2—3个主导产业和首位产业，提升产业首位度和竞争力。建设小微产业园20个，力争实现产值50亿元。鼓励开发区实施“标准地块”，提高亩均投资强度和产出。

（二）坚持融入长三角一体化，提升开放合作水平。落实《长三角一体化发展三年行动计划》和《G60科创走廊总体发展规划3.0版》，加强区域间互联互通、互促互融，打造内陆开放新高地。

促进区域合作更深入。深化与上海及南京、杭州、合肥都市圈城市合作。加强与G60科创走廊城市融合发展，组建G60电机产业创新联盟。力争长三角产业合作示范区核心区建设落在宣城，开工落地苏皖合作示范区、松江·宣城产业园、宣城·萧山产业园等一批重大区域合作项目。精心办好第九届文房四宝文化旅游节、皖东南名品展销会等，努力打造高端展会品牌，提升宣城美誉度和影响力。

推动招商引资更有效。健全完善市直招商引资工作机制，建立全流程工作推进机制，实行双月调度、季度核查制度，强化考核奖惩。优化整合招商机构，配强专业招商队伍，加大精准招商力度。创新招商方式，建立常态化以商招商机制，开展社会化招商试点，拓展招商渠道。突出园区招商主体作用，制定重点产业招商目录，运用“产业+基金+政策+平台”等招商新模式，突出产业链招商，力争引进央企、民企、外企等龙头企业和重大项目取得新突破。确保开工竣工投产项目数和形成固定资产投资额均增长10%以上，实际利用外资增长6.5%以上。

促进外贸增长更稳定。拓展与“一带一路”沿线国家和地区合作，组织企业参加境外会展，扩大进出口贸易，推动出口市场多元化。大力培育外贸主体，新增进出口实绩企业90户以上。加强出口基地建设，扩大机电设备、关键零部件及日用消费品进口规模。申报设立二类口岸，筹建宣城综合保税区。打通铁海联运、江海联运通道，推进关检业务深入融合。鼓励企业ERP与海关联网，推动无缝报关放行。

实现开放通道更便捷。编制宣城地区铁路枢纽总图规划。全面开工建设宣绩高铁，加快商合杭、皖赣铁路扩能改造宣城段、巷口桥铁路物流基地和芜宣机场建设，积极推进宁宣、宣镇、杭临绩等高铁和宣城至芜宣机场至高淳轨道交通、绩溪通用机场等项目前期工作。完成交通建设投资50 亿元以上，开工宣泾高速部分工程，加快芜黄高速宣城段建设，推进申嘉湖高速西延宁国段、宣广高速改扩建、扬绩高速黄山连接线、宁杭高速二通道等项目前期工作。实施 16个国省道项目建设，建成一级公路3条。开通水阳江航道和定埠港，实现宣州综合码头一期开埠、二期开工。

（三）坚持深化改革创新，持续增强发展动力活力。坚持改革引领、创新驱动，大力支持民营经济发展。

深化供给侧结构性改革。推进闲置土地、僵尸企业、僵尸项目“三清”工作，破除无效供给。完善多主体供给、多渠道保障、租购并举住房制度，促进房地产市场平稳健康发展。加强建筑市场监管，做大做强建筑业。落实国家更大规模的减税降费政策，继续实施减税清费降本行动，切实减轻企业负担。加大基础设施等领域补短板力度，推进重点项目建设，力争实施市重点项目600个以上，完成投资600亿元以上，新开工和竣工重点项目400个以上，谋划储备项目600个以上。严格落实“四督四保”制度，确保列入省亿元以上项目投资完成率、开工率、竣工率继续保持全省前列。

加大科技创新力度。创成省级创新型城市，启动国家创新型城市创建。建成宛陵科创城一期，确保已签约机构入驻运行，完善体制机制，打造“众创空间+孵化器+加速器”创新创业链条。加强高新技术企业培育，全年新认定高新技术企业100户以上，高新技术产业增加值增长15%以上。对接产学研合作项目300项以上，新增省级以上企业创新平台15家以上，力争全社会研发投入强度达1.7%以上，每万人口发明专利拥有量达10件以上。深入实施“宛陵聚才行动”，引进高层次人才团队10家以上。继续实施“宣城籍优秀人才回归工程”。加大对中小企业创新支持力度，加强知识产权保护和运用，形成有效的创新激励机制。实施质量品牌提升工程，新申请商标注册4000件，新申报中国驰名商标3件、马德里国际商标4件、地理标志证明商标3件。

做大做强民营经济。常态化开展“四送一服”双千工程，落实国家和省、市支持民营经济发展政策措施，真心实意帮助民营企业解决难题，提振民营企业家信心。落实市场准入负面清单制度,持续深化商事制度改革，完善“多证合一”改革，全面推开“证照分离”，力争新登记民营企业3600户、个体工商户1.4万户以上。统筹用好市民营经济（中小企业）发展专项资金，发挥资金效应。搭建银企对接平台，推动金融更好服务实体经济，引导信贷资金更多支持民营和小微企业发展。加大融资担保支持力度，放大担保功效。力争新增贷款130亿元以上、新型政银担贷款50亿元、税融通贷款10亿元。实施“助企翱翔”计划，积极对接上交所科创板，新增企业上市（挂牌）10家，完成直接融资80亿元以上。推进技工强市建设，搭建劳动者就业需求和企业用工信息对接平台，加强跨地区劳务对接，常态化开展“2+N”招聘活动，缓解企业用工困难。弘扬企业家精神，开展明星企业、功勋企业家和宣城工匠评选表彰。建立健全民营企业保护机制，构建亲清新型政商关系。

（四）坚持区域协调发展，加快中心城市建设。健全协调联动发展机制，加强“一区两廊”建设，提升区域发展水平。

下大力气加快市区发展。深化市区一体化发展，支持宣州区在综合百强区中争先进位。加快宣城经开区体制机制创新，确保主要经济指标增速领先全市，力争园区总产值突破100亿元、规上企业突破100户；强化招大引强，力争引进5亿元以上项目10个、10亿元以上项目5个，亿元以上项目到位省外资金突破100亿元；推进机械与装备制造、新能源新材料、食品加工、生物医药、软件大数据等产业集群培育；实施百个项目落地攻坚行动，加快锦美碳材、普丽盛食品装备制造、亚德林高端装备制造等项目建设。完善宣城现代服务业产业园区管理机制和财税体制，推进市场化运营，深入实施“五个百亿”工程，力争新开工项目10个，固定资产投资增长10%；羽绒产业园一期项目全部建成投产，力争实现产值60亿元，二期项目开工建设；启动智能制造产业园建设，加快电子商务与快递业融合发展集中区建设；整合农批、冷链、物流等市场资源，打造长三角农产品供应链中心。宣城高新区力争创成国家级高新区和知识产权试点园区，做大做强新一代信息技术、新材料、智能制造和生物技术产业。

提升中心城市能级和品质。坚持“双百”城市总规引领，适时推进行政区划调整，壮大城市规模。持续抓好全国城市设计试点工作，扎实推进省级城市生态修复、功能修补试点。按照“上急需、保基本、补短板、提品质、有保障、能平衡”原则，推进城区“十大板块”建设，实施城市建设项目183个，完成投资150亿元以上。继续化解城建历史遗留问题。进一步完善城区路网，新建市政道路项目14个以上、建成32个。推进高铁两站广场建设，开通宣城客运总站。创建省级“公交示范市”，推进市区公交发展和公共停车场建设。加快“533”社会事业补短板工程建设，确保北师大宣城学校秋季正式招生，完成省立医院皖南妇幼分院、皖南医药卫生学校一期主体工程，开工建设皖南中医院、市第五中学、市委党校新校区、老干部和职工活动中心。启动水阳江两岸综合保护与利用规划。实施城市水生态体系建设。将城市应急备用水源建设作为首要工程强力推进。全面完成城市防洪排涝重点工程，确保市区防洪、排涝标准明显提高。不断提升全国文明城市、卫生城市建设水平，强化城市精细管理，持续开展“城市六乱”治理，解决菜市场、餐饮油烟、市容秩序等突出问题。研究制定城市规划区农民建房办法。推进城市生活垃圾强制分类，确保市生活垃圾焚烧发电二期投入使用，完成建筑垃圾处置场综合治理，启动餐厨垃圾处置项目和医疗固废产业园建设。加快“智慧城管”建设，深化城市执法体制改革，提高城市综合治理水平。

支持县域经济做大做强。因地制宜实施县域分类考核评价，促进县市区高质量发展。加快推进宣郎广宁智创走廊建设，支持广德撤县设市，郎溪、广德对标全国百强县，力争宁国位次前移。鼓励有条件的县（市）建设全国创新型县（市）。加快泾旌绩文化旅游生态健康产业走廊建设，支持泾县申创国家中小型电机智能制造示范、电机绿色供应链项目，支持绩溪争创全省县域特色产业集聚发展基地，支持旌德加快全国“两山”实践创新基地建设。加快建设经济强镇，新增省级特色小镇2个。

（五）坚持农业农村优先发展，扎实推进乡村振兴战略。建立统筹推进的运行机制，开展“十镇百村”示范创建，推动乡村振兴战略深入实施。

提升现代农业水平。稳定粮食生产，加快发展特色优势产业，增加优质绿色农产品供给。实施农业产业化提升行动，支持龙头企业发展，力争农产品加工产值达960亿元。加快农业园区建设，提升产业集聚度。加强农产品质量安全监管，打响区域公用品牌，争创“三品一标”产品35个。持续抓好非洲猪瘟疫情防控，加快家禽、生猪产业转型升级。培育新型农业经营主体，争创省级示范合作社6个、家庭农场15个、产业化示范联合体7个。解决小农户生产经营面临的困难，推动小农户与现代农业有机衔接。加强新型职业农民培育，培养一支懂农业、爱农村、爱农民的“三农”工作队伍。

持续深化农村改革。全面完成全国农村集体产权制度改革试点任务。推进国家集体林权改革试验示范区建设。全面实施农村“三变”改革，着力破除体制机制障碍。深入推进“两权”抵押贷款试点。探索农村宅基地“三权分置”改革。继续做好“大棚房”问题排查整治。探索建立涉农资金统筹整合长效机制。扶持村集体经济发展，增强“造血”能力。

补齐农村基础设施短板。推进港口湾水库灌区、牛岭水库、扬溪源水库等重点工程建设，继续实施水利灾后薄弱环节建设治理工程，加快水阳江中游治理等重大项目前期工作。加强城农网专项工程建设。推行“路长制”，加强“四好农村路”建设，完成农村道路扩面延伸工程400 公里。

加强美丽乡村建设。完成49个省级中心村建设任务、新申报56个，建设市县级中心村60个左右。以“三大革命”和“五清一改”为抓手，全面推进农村人居环境整治，力争走在全省前列。推进农村垃圾源头分类减量，提升农村生活垃圾无害化处理率。以新时代文明实践中心建设为载体，活跃农村文化，培育乡风文明。

（六）坚持生态优先、绿色发展，积极打造生态文明样板。深入践行习近平生态文明思想，推进安徽（宣城）绿色发展创新实验区建设，打造水清岸绿产业优美丽长江（宣城）经济带。

着力加快生态保护与修复。坚决贯彻落实习近平总书记重要指示批示精神，认真抓好扬子鳄自然保护区突出问题整改工作，确保扬子鳄野外种群得到有效保护和发展。坚持举一反三，切实加强自然保护区管理。高质量推进中央环保督察和“回头看”反馈问题整改，确保改彻底、改到位。启动生态保护红线勘界立标工作，落实各项管控措施。进一步加强南漪湖、敬亭山区域生态保护。开展增绿增效行动，完成营造林111.5万亩。落实最严格的水资源管理和耕地保护制度。推动形成人与自然和谐发展，还自然以宁静、和谐、美丽。

着力完善生态文明制度体系。坚持共抓大保护、不搞大开发，落实主体功能区规划。健全资源有偿使用和补偿机制，深入实施水环境、空气质量生态补偿机制。推深做实河（湖）长制、林长制，开展河湖岸线专项规划和划界工作。完善生态环境监管体系，加强企业环境监管，实施生态环境损害赔偿制度，让制度成为刚性约束和不可触碰的高压线。

着力发展壮大现代服务业。服务业是宣城发展的短板，也是发展潜力所在。大力发展生产性服务业，积极引进区域性第三方物流企业落户我市，培育限上服务业企业，争创省级现代服务业集聚区,力争服务业增加值增长8.5%左右。推进“电商宣城”建设，力争年度网络交易额增长20%以上。发展壮大文房四宝产业，申创省级文化产业园区（基地）。持续开展国家全域旅游示范区创建，确保接待游客数、旅游总收入增长16%和20%以上。加快推进新港文旅综合体、宣酒小镇等项目。加强旅游景区基础设施、服务设施建设，出台农家乐（民宿）管理办法，建成市县旅游集散中心。精心打造皖南川藏线，提升敬亭山“江南诗山”品牌形象。

（七）坚持攻坚不停步，奋力打好三大攻坚战年度战役。制定实施年度战役计划，针对突出问题，打好重点战役，确保三大攻坚战取得新成效。

防范化解重大风险。构筑常态化的金融风险监测、排查和处置机制，深入开展互联网金融风险专项整治，严厉打击非法金融行为，维护地方金融安全。加强政府性债务规范管理，稳妥处理地方政府债务风险，确保不违规新增债务。加强国有资产监管，推进国有平台公司市场化转型，培育拓展主业，完善企业内控体系，全面加强风险管理，做强做优做大国有资本。

提升精准脱贫质量。坚持问题导向，重点解决好实现“两不愁三保障”面临的突出问题，研究解决收入水平略高于建档立卡贫困户的群体缺乏政策支持等新问题，高质量完成年度脱贫攻坚任务。扎实推进脱贫攻坚“十大工程”,打造产业扶贫、就业扶贫升级版。规范扶贫小额信贷发放，全面落实健康扶贫、教育助学等政策，统筹各类兜底保障措施。构建防范返贫、稳定脱贫长效机制，同步推进非贫困村脱贫和困难群众帮扶工作。注重扶贫同扶志、扶智相结合，提升贫困群众脱贫能力。

打好污染防治攻坚战。推进大气污染综合治理攻坚行动，持续开展“散乱污”企业整治，加强煤炭消费总量控制和烟尘综合治理，开展工业炉窑和挥发性有机物专项整治，推进柴油货车污染治理攻坚。加强重点河流湖泊、集中式饮用水源地和城市黑臭水体治理，持续开展入河排污口和排放口规范化整治，不断改善水质环境。强化农业面源污染防控，提升畜禽养殖废弃物资源化利用和秸秆综合利用水平，严格固体废弃物监管，严防土壤环境风险。

（八）坚持以人民为中心，加快民生社会事业发展。以造福人民为最大政绩，不断满足人民日益增长的美好生活需要，持续增强群众获得感、幸福感、安全感。

加强就业和社会保障。实施就业优先战略和积极就业政策，促进创业就业，重点解决好高校毕业生、农民工、退役军人等群体就业。稳步推进城乡低保统筹，完善社会救助制度，提高城乡低保标准和特困人员供养基本生活标准。实施42个老旧小区整治。完成5365套棚户区改造和1456户农村危房改造任务。加强城乡社区（村）儿童之家、老年人日间照料中心和残疾人之家建设。加强未成年人保护。建成市精神卫生社会福利中心。深化殡葬改革，推进公益性公墓建设。发展社会福利和公益慈善事业。

促进教育和文化事业发展。实施市区中小学幼儿园建设与发展三年行动计划，新建小学3所、初中2所，建成市体校。继续实施第三期学前教育行动计划，全市新改建公办幼儿园12所。加强义务教育学校标准化建设，巩固义务教育均衡发展成果。推动高中阶段教育全面普及和发展提升。提升中等职业教育质量。大力支持合工大宣城校区、宣城职业技术学院建设发展。鼓励支持和规范民办教育发展，巩固提高校外培训机构专项治理成果。加强教师队伍建设，完善落实教师待遇保障机制。持续开展群众性精神文明创建活动。建设乡镇、村级综合文化服务中心，打造农家书屋、全民阅读等文化事业品牌。加强历史文化名城名镇名村保护，推进市档案馆新馆建设。

加快健康宣城建设。深化医药卫生体制改革，巩固国务院大督查表彰成果，开展建立现代医院管理制度工作，启动县级医院医疗服务能力提升工程，加强市县公立医院重点学科建设，推进乡村医疗服务能力和体系建设。支持广德、绩溪、旌德创建国家卫生县城。完善计生和妇幼配套政策。开展全民健身品牌赛事和“一县一品”群众体育品牌活动。举办市第二届运动会。加快市体育中心建设，建成使用游泳馆、全民健身馆。

加强平安宣城建设。深入开展扫黑除恶专项斗争，大力实施“雪亮工程”、科技强警工程，推进“平安细胞”创建，持续开展“守护平安”系列行动，依法打击和惩治各类违法犯罪行为。认真落实安全生产责任制，扎实推进“铸安”行动，抓好重点行业领域专项整治和隐患排查治理，确保安全生产形势持续稳定。巩固省级食品药品安全城市创建成果。加强气象、水文、地震、红十字会等工作。健全应急管理体制，加强应急救援能力建设，提升综合防灾减灾救灾能力。

提升社会治理水平。持续推进法治宣城建设，加大“七五”普法力度，扩大法律援助范围，争创全省法治文化建设示范市。深化基层综治中心建设，创新矛盾纠纷“七大调解法”，开展标准化调解委员会创建。纵深推进人民满意信访窗口创建，完善领导干部开门接访制度，依法及时解决群众合理诉求。继续探索“党建+三治”乡村治理模式，构建城市社区“阳光议事厅”治理平台，加强社会心理服务体系建设。持续推进社会诚信体系建设。支持工会、共青团、妇联等群团工作。做好民族宗教、外事侨务、对台等工作。高质量完成第四次全国经济普查工作，推进第三次全国国土调查工作。加强双拥共建和优抚安置工作，争创新一轮全国双拥模范城。强化国家安全、全民国防教育和国防动员、人民防空工作，推进军民融合深度发展。做好退役军人服务管理工作，维护军人军属合法权益，切实让军人成为全社会尊崇的职业。

坚持群众无小事、民生需求是大事，在认真总结省定民生工程和市政府民生实事经验做法的基础上，市政府今年还将办好10项民生实事：①市区失能半失能老人家庭护理人员免费技能培训；②对城区“三无”对象中的特困供养人员发放居家养老护理补贴；③市区住宅小区把式车辆停车棚建设；④市区保障性蔬菜基地能级提升工程；⑤全市新生儿遗传代谢性出生缺陷疾病干预救助；⑥市区住宅小区老旧电梯安全评估；⑦新建、改造提升市区公厕；⑧全市特困妇女“两癌”免费筛查；⑨市区部分菜市场升级改造；⑩市区环卫工人“爱心驿站”建设。

三、全面加强政府自身建设

打铁必须自身硬。我们将坚持以人民为中心，担当尽责、真抓实干，建设人民满意的服务型政府。

对党绝对忠诚。学懂弄通做实习近平新时代中国特色社会主义思想和党的十九大精神，树牢“四个意识” ,坚定“四个自信”,坚决做到“两个维护”。把旗帜鲜明讲政治落实到政府工作各方面、全过程。严守政治纪律和政治规矩，确保党中央国务院、省委省政府和市委各项决策部署落实到位。高标准完成省委巡视反馈意见整改工作，做到动真碰硬、见底清零，举一反三、标本兼治。

坚持依法行政。带头尊法学法守法用法，积极运用法治思维和法治方式深化改革、推动发展、化解矛盾、维护稳定。健全政府立法机制，开展立法实施后评估工作，提高政府法治工作水平。严格遵守重大行政决策公众参与、专家论证、风险评估、合法性审查、集体讨论决定的法定程序，提高决策科学化、民主化、法治化水平。深化行政综合执法改革，规范执法行为。自觉接受市人大及其常委会的法律监督、工作监督和市政协的民主监督，依法执行市人大决议决定，高质量办理人代会议案、人大代表建议、政协委员提案和人大常委会审议意见、政协资政协商建议。全面推进政务公开，完善政府守信践诺机制，让权力运行更加阳光透明。

提升施政本领。加强学习型政府建设，全面增强“八种本领”。加强调查研究、深入细致谋划，创造性贯彻落实上级决策部署。充分发挥市场在配置资源中的决定性作用，更好发挥政府作用，提升经济工作能力和水平，争做“精明的经济工作者”。坚持经济运行和重点工作调度、重大项目建设观摩、季度集中开工竣工投产、“无会周”外出招商等制度，完善工作推进机制，强化跟踪问效，确保事事有回音、件件有着落。

优化政务服务。深入推进“放管服”改革。深刻转变政府职能，改革机构设置，优化职能配置，完成市县政府机构改革。深化“互联网+政务服务”，推进“一网一门一次”改革和“一网通办、不见面审批”，打响“马上就办、真抓实干”服务品牌，打造“四最”营商环境。推行并联审批、限时办结、超时默认制度。推进“双随机”跨部门联合抽查。强化互联网思维，推进政务信息资源整合和政务数据互通共享。优化“12345”政府服务热线运行，提高事项办结率、问题化解率。

强化实干担当。坚持崇尚实干、力戒空谈，敢于直面矛盾、勇于担当尽责，主动往前站、随我上、带头干，争当实干兴宣的行动英雄。践行“一线工作法”，强化工地情结，用脚丈量工作，现场解决问题。坚持开短会、讲短话、发短文，大幅度减少会议和文件，腾出更多时间、精力抓落实。激励干部担当作为，增强干事创业激情，完善激励约束机制、容错纠错机制，为担当干事者鼓劲撑腰，对懒政怠政者严肃问责。

加强廉政建设。深入贯彻全面从严治党新要求，始终把政治建设摆在首位，认真落实主体责任，切实履行“一岗双责”，扎实推进政府系统党风廉政建设。认真落实中央八项规定及实施细则精神和省、市委有关规定，加强预算管理，强化审计监督，严格公车使用，严控“三公”经费。持之以恒正风肃纪，坚决破除形式主义、官僚主义。严格规范督查检查考核，严控总量和频次，避免微观执法简单化、“一刀切”。支持纪检监察机关履行职责，坚决查处侵害群众利益的不正之风和腐败问题，做到干部清正、政府清廉、政治清明。

各位代表，同志们！山再高，往上攀，总能登顶；路再长，走下去，定能到达。让我们更加紧密地团结在以习近平同志为核心的党中央周围，在省委、省政府和市委的坚强领导下，不忘初心、牢记使命，勇于担当、搏击奋进，为开创皖苏浙省际交汇区域中心城市新局面、争当长三角一体化高质量发展安徽排头兵、谱写宣城人民美好生活新篇章不懈奋斗!
